# Late-Term Elective Abortion and Susceptibility to Posttraumatic Stress Symptoms

**DOI:** 10.1155/2010/130519

**Published:** 2010-08-01

**Authors:** Priscilla K. Coleman, Catherine T. Coyle, Vincent M. Rue

**Affiliations:** ^1^Human Development and Family Studies, Bowling Green State University, Bowling Green, OH 43403, USA; ^2^Alliance for Post-Abortion Research Training, Madison, WI 53711, USA; ^3^Institute for Pregnancy Loss, 3030 Hartley Road, Suite 220, Jacksonville, FL 32257, USA

## Abstract

The primary aim of this study was to compare the experience of an early abortion (1st trimester) to a late abortion (2nd and 3rd trimester) relative to Posttraumatic Stress Disorder (PTSD) symptoms after controlling for socio-demographic and personal history variables. Online surveys were completed by 374 women who experienced either a 1st trimester abortion (up to 12 weeks gestation) or a 2nd or 3rd trimester abortion (13 weeks gestation or beyond). Most respondents (81%) were U.S. citizens. Later abortions were associated with higher Intrusion subscale scores and with a greater likelihood of reporting disturbing dreams, reliving of the abortion, and trouble falling asleep. Reporting the pregnancy was desired by one's partner, experiencing pressure to abort, having left the partner prior to the abortion, not disclosing the abortion to the partner, and physical health concerns were more common among women who received later abortions. Social reasons for the abortion were linked with significantly higher PTSD total and subscale scores for the full sample. Women who postpone their abortions may need more active professional intervention before securing an abortion based on the increased risks identified herein. More research with diverse samples employing additional measures of mental illness is needed.

## 1. Late-Term Elective Abortion and Susceptibility to Posttraumatic Stress Symptoms

Considerable research attention over the past several decades has focused on isolating the physical and psychological effects of induced abortion [1–5]. The majority of abortions in the United States are performed early in pregnancy and most of the research pertaining to indicators of postabortion health has logically involved the study of women who have undergone 1st trimester abortions. However, it is significant to note that 12%-13% of the annual 1.2 million U.S. abortions are performed after the first trimester [6–8] and this translates out to approximately 144,000 per year, with 3.7% or 36,000 taking place at 16–20 weeks and 1.3% or 15,600 occurring beyond the 20th week of pregnancy. 

Although empirical data is in short supply, a few large scale research efforts have revealed that 2nd trimester (13–24 weeks) and 3rd trimester (25–36 weeks) abortions pose more serious risks to women's physical health compared to 1st trimester abortions [9, 10]. The abortion complication rate is 3%–6% at 12-13 weeks gestation and increases to 50% or higher as abortions are performed in the 2nd trimester [9]. Moreover, using national U.S. data spanning the years from 1988 to 1997, Bartlett and colleagues [10] reported the following rates of abortion-related mortality: 14.7 per 100,000 at 13–15 weeks of gestation, 29.5 per 100,000 at 16–20 weeks, and 76.6 per 100,000 at or after 21 weeks. 

Based on the potential for serious consequences associated with late-term abortion, a first step toward reducing the numbers of late-term abortions is to gain a comprehensive understanding of why women decide to terminate as opposed to continue a pregnancy once they have allowed the pregnancy to progress for several months. According to the Guttmacher Institute [11], the most frequently endorsed reasons for late-term abortions include the following: (1) not realizing one is pregnant (71%), (2) difficulty making arrangements for an abortion (48%), (3) fear of telling parents or a partner (33%), and (4) feeling the extended time is needed to make the decision (24%). In the Guttmacher study, only 8% of the women sampled indicated pressure not to have an abortion from someone else was part of the reason for delay and fetal abnormalities were identified as factoring into only 2% of all late-term abortion decisions.

Researchers have identified several differences between women who obtain early versus late abortions. For example, decision ambivalence is often characteristic of women who undergo abortions in the 2nd trimester and beyond [12–14]. Further, women who obtain 2nd trimester abortions have reported more deficient social supports and more energy expended toward assessing the resources available to help them keep a child compared to women who obtain 1st trimester abortions [14, 15]. Research suggests that 30% of women who delay an abortion beyond 16 weeks are afraid to tell those closest to them about the pregnancy [11]. When compared to women obtaining earlier abortions, women who obtain late-term abortions are more likely to experience stronger attachment to the fetus, have more moral or religious objections to abortion, and concede to an abortion based on the wishes of others [15, 16]. Finally, women who seek late-term abortions (after 16 weeks) are significantly more likely to be under age 18, Black, unemployed, and/or poor [11]. 

As indicated above there is not an extensive published literature on the physical effects of late abortions; however there are even fewer published studies on women's mental health outcomes after 2nd trimester abortions. Nevertheless, it is logical to anticipate more serious mental health problems in response to abortions occurring later in pregnancy compared to earlier terminations for various reasons: (1) awareness that the fetus has developed more fully prior to the termination, (2) women have more opportunity to bond with the developing fetus, (3) there may be more active desire to maintain the pregnancy, and/or (4) pressure from others to abort may be more pronounced. In fact, most of the established predictors of late-term abortion described above, including decision ambivalence and dissatisfaction, lacking support to carry to term, a strong attachment to the fetus, timing during adolescence, and low income are predictors of poor postabortion psychological adjustment in the general abortion literature [17–22]. 

In a study of British women who had prostaglandin-induced abortions between 20–24 weeks gestation and felt fetal movements, 25% reported being depressed after the procedure [23]. Further, Söderberg et al. reported that 37.5% of women who underwent 2nd trimester abortions experienced “extreme postabortion emotional problems” [24]. Although these studies indicate that late-term abortions are more likely to initiate psychological problems, they were both very small scale and did not involve direct comparisons to women undergoing 1st trimester abortions with logical controls for variables likely to discriminate between the two populations.

The lifetime prevalence of Posttraumatic Stress Disorder (PTSD) for U.S. women is approximately 13% [25]. Empirical evidence of a link between 1st trimester abortion and PTSD symptoms has accumulated in recent years [26–30]. In fact 12–20% of women with an abortion history meet the full diagnostic criteria for PTSD with considerably higher percentages of women experiencing some trauma symptoms, while not meeting the full criteria [28–30]. Even when the full criteria are not met, the more PTSD symptoms present, the greater the risk of psychological impairment and suicidal ideation [31]. In the current study, comparisons were made between women who had an early elective abortion (up to 12 weeks gestation) and women who had undergone a later elective abortion (13 weeks gestation or later) based on individual PTSD symptoms, PTSD symptom subscale scores (Intrusion, Avoidance, and Hyperarousal), total PTSD scores, and the degree to which PTSD symptoms met the full DSM-IV diagnostic criteria. 

As an anxiety disorder, PTSD is initiated by exposure to a psychosocial stressor which is perceived to be traumatic. This disorder is comprised of three stressor-related criteria not present before the trauma: (1) *Intrusion* which involves persistent and unwanted reexperiencing of the traumatic event in the form of recurrent and distressing memories, flashbacks, and hyperreactivity to associated stimuli; (2) *Avoidance* which pertains to persistent and deliberate efforts to avoid recalling the traumatic event using various forms of denial, dissociation or detachment; and (3) *Hyperarousal* which is a general uneasiness or jumpiness that may include insomnia, the tendency to startle easily, feelings of impending danger or disaster, trouble concentrating, extreme irritability, and possibly violent behavior.

Although no previous studies have been published comparing the mental health of women undergoing early and late term abortions, the evidence reviewed above is sufficient to hypothesize that abortions occurring across the 2nd trimester and into the 3rd trimester would be associated with higher levels of PTSD symptomatology than 1st trimester abortions. Since 2nd and 3rd trimester abortion are less common than 1st trimester abortions and women may be more reticent about acknowledging such abortions due to stronger social prohibitions against later terminations, web-based data collection was deemed a useful method in that it affords a high level of anonymity. The obvious drawback to this methodology is the risk for selection bias wherein more women who have struggled with an abortion experience may be more inclined to participate due to the increased salience of the experience and a possible quest for answers. 

Established benefits of internet data collection include time and cost efficiency [32, 33], access to difficult to reach populations [34, 35], and enhancement of participant comfort and engagement [36, 37]. A review by Skitka and Sargis [38], indicated that as early as the years 2003 to 2004, 21% of APA journals had published at least one study with online data collection, suggesting this is rapidly becoming an established mode of data collection. The most frequently cited criticism of web-based surveys is that they are comprised of convenience samples, rendering generalization difficult [39, 40]. However, even this shortcoming engenders benefits such as clarity and thorough responses that are less inclined to be contaminated by social desirability biases and underreporting [41–43]. Several published papers indicate that online data collection is equivalent to more traditional data collection methodologies in terms of reliability, validity, and representativeness [44–47].

The primary goal of the present exploratory study was to compare the mental health status of a sample of women who experienced a 1st trimester abortion (up to 12 weeks gestation) to women who had a 2nd or 3rd trimester abortion (13 weeks and beyond). In the analyses, sociodemographic and personal history factors, particularly those related to significant life stressors such as exposure to abuse, that may systematically vary across the two groups (early and late) were controlled in order to more accurately examine the independent effects of abortion timing. A secondary goal was to provide additional descriptive data on women who delay abortion decisions until the 2nd and 3rd trimesters with a focus on variables pertaining to the abortion decision.

## 2. Method

### 2.1. Procedure

Surveys were posted on an internet website from April, 2005 through August, 2008. Although the time frame was chosen for convenience, the goal was to collect data until a minimum of 50 women who experienced a late-term abortion had responded in order to insure adequate statistical power. All respondents who had experienced an abortion and completed at least 95% of the items on the survey were included. Participants were informed that submission of the survey constituted consent to participate and they were told they could withdraw at any point. Informational website links offering support or counseling services were provided for interested participants. Recruitment occurred through email requests to US-based crisis pregnancy centers and to a few additional organizations that offered postabortion counseling. Finally, interested participants could find the survey simply by using internet search engines.

### 2.2. Measures

Questions comprising the survey addressed five sociodemographic characteristics (age, race, education, marital status, and number of children), meaningfulness of religious affiliation, abortion history, reasons for abortion, perceived adequacy of preabortion counseling, partner agreement in abortion decision-making, political opinion regarding abortion at time of procedure, relationship status with the partner, mental health history, abuse history, trauma symptoms related to abortion, abortion-related anger, relationship problems, sexual problems, and general stress attributed to abortion. The majority of items measuring demographic characteristics, personal history, and the circumstances surrounding the abortion were dichotomous (yes/no). The precise nature of the items and response options are easily identified by the data in Tables 1 and 2.

Posttraumatic Stress Disorder symptoms were assessed with the PTSD Checklist-Civilian Version (PCL-C) [48]. The PCL is a 17 item index of the presence of PTSD symptoms. For the purposes of the present investigation, a score of “1” was entered for each item endorsed on the scale at the level of “moderately”, “quite a bit”, or “extremely” yielding a total score range from 0–17. If the respondents indicated “not at all” or only “a little bit” she was not identified as having the corresponding symptom. Subscale score ranges were from 0 to 5 for Intrusion or reexperience (5 items), 0 to 7 for Avoidance (7 items), and 0 to 5 for Hyperarousal (5 items). Respondents were considered to have met the symptom criteria for a diagnosis of PTSD based on DSM-IV criteria: (1) one or more endorsements of intrusion, (2) three or more endorsement(s) of avoidance symptoms, and (3) two or more endorsements of hyperarousal symptoms not present prior to the abortion. Reliability and validity evidence for the PCL is provided by Weathers and colleagues [48]. With the current sample, internal consistency reliability estimates using Cronbach's alpha for the full scale and for the Intrusion, Avoidance, and Hyperarousal subscales were equal to .92, .82, .80, and .82, respectively.

### 2.3. Statistical Analyses

All analyses were conducted using SPSS software and included both basic descriptive statistics and inferential statistical tests to examine the hypotheses and conduct secondary tests of the data. Specific analyses conducted included independent *t*-tests, analyses of variance (ANOVA), analyses of covariance (ANCOVA), multivariate analyses of variance (MANOVA), multivariate analyses of covariance (MANCOVA), and logistic regression. In the analyses wherein statistical controls were employed, the following variables were entered: race, marital status at the time of the abortion, number of years of formal education, number of abortions, number of years since the target abortion, having received mental health counseling before the abortion, having been hospitalized for emotional problems before the abortion, meaningfulness of the respondent's religion, and a childhood or adult history of physical or sexual abuse.

## 3. Results

### 3.1. Sample Characteristics

Surveys were completed by 374 women with 81% of the respondents indicating U.S. citizenship. Additional respondents identified the following countries of citizenship: England (4%), Canada (6.4%), and Australia (2.7%), with smaller percentages from France, Ireland, Norway, Romania, Czechoslovakia, Germany, Sweden, New Zealand, South Africa, Kenya, Mexico, Nicaragua, Brazil, Nepal, and South Korea. The average age of the respondents was 38 years at the time the survey was completed (SD = 11.1). Also, at the time of the survey, 48.0% were married for the first time, 10.5% were married for the second time, 12.1% were divorced and single, 2.2% were separated, and 26.4% had never married. Religious affiliations were indicated to be 81.6% Christian, 3% Jewish, 9.5% other, and 8.6% none at the time of the survey. Most of the sample endorsed liberal views of abortion at the time of the procedure, with 24% believing abortion should be legal for any reason throughout pregnancy and an additional 36% contending abortion should be available for any reason during the 1st trimester. Only 24% felt it should be legal under various circumstances and 16% believed it should never be legal. 

Approximately 14% (*n* = 52) reported having undergone an abortion between 13 and 30 weeks gestation (M = 16.87 weeks; SD = 4.24) and 86% reported abortions up to 12 weeks gestation (M = 8.23 weeks; SD = 2.39). No abortions beyond 30 weeks were reported. The women reported an average of 15 years (SD = 11.8) had passed since the abortion and again no significant differences were observed between the early and late groups.

### 3.2. Additional Demographic and Control Variables

Table 1 provides the full sample frequencies for respondents' race, education, marital status, number of children, number of abortions, meaningfulness of religion, mental health counseling prior to the abortion, hospitalizations for emotional reasons before the abortion, and the experience of physical and sexual victimization in childhood and adulthood. Use of independent *t*-tests and logistic regression analyses revealed no significant differences between the early and late abortion groups relative to age, ethnicity, marital status at the time of the abortion, religious orientation, meaningfulness of religion, marital status at the time the survey was completed, education, number of children, number of abortions, mental health counseling prior to the abortion, preabortion hospitalizations for emotional problems, and the experience of sexual abuse in childhood or adolescence. However, the women who obtained late abortions were more likely to report having been the victim of physical abuse in childhood (*t* (373) = −2.37, *P* < .001), to have been the victims of physical abuse in adulthood (*t* (273) = −2.05, *P* = .044), and to have been the victim of sexual abuse in adulthood (*t* (373) = −2.94, *P* = .005). 

Table 2 provides data regarding the percentage of study respondents from the early and late abortion groups who indicated agreement with various parameters surrounding the abortion decision. As indicated by the data presented, logistic regression analyses revealed that women who had experienced a late-term abortion were significantly more inclined to report the following: (1) the pregnancy was desired by their partner: (2) someone other than their partner pressured the decision: (3) the respondent left her partner before the abortion: (4) the respondent's partner did not know about the abortion until afterwards: and (5) physical health concerns factored into the abortion decision. In contrast, women who secured earlier abortions were more likely to report abortion decision agreement with their partner and they were more likely to report that mental health, financial, and educational concerns factored into their decision to abort.

### 3.3. Testing of the Hypothesis

In order to test the hypothesis that 2nd and 3rd trimester abortions would be more stressful than 1st trimester abortions, several analyses were conducted that enabled the researchers to determine if women experiencing late-term abortions are generally more at risk for PTSD symptoms. A MANOVA was conducted using timing of the abortion (early versus late in pregnancy) as the independent variable and the three subscales of the PCL as the dependent variables. The multivariate effect was significant, *F* (3,322) = 2.69, *P* = .046. In addition, the univariate effect for the Intrusion subscale was significant, *F* (1,324) = 7.49, *P* = .007, with the means for the early and late abortion groups equal to 2.42 (SD = 1.63) and 3.13 (SD = 1.64), respectively. Similarly, the difference between the two abortion timing groups was significant for the Hyperarousal subscale, *F* (1,324) = 4.76, *P* = .030, with the mean for the early group lower (M = 2.22; SD = 1.78) than the mean for the late group (M = 2.85; SD = 1.95). However the univariate effect for the Avoidance subscale was not significant. The variables in this first analysis were then entered into a MANCOVA with controls for several demographic and personal experience variables (race, marital status at the time of the abortion, number of years of formal education, number of abortions, number of years since the target abortion, having received mental health counseling before the abortion, having been hospitalized for emotional problems before the abortion, meaningfulness of the respondent's religion, and a childhood or adult history of physical or sexual abuse). The results revealed only one significant univariate effect, for the Intrusion Subscale, *F* (1, 260) = 4.91. *P* = .026. The adjusted mean for the early group was equal to 2.51 (SE = .103) and the adjusted mean for the late group was equal to 3.15 (SE = .26). 

An ANOVA was conducted with abortion timing groups again employed as the independent variable and total PCL scores as the dependent variable. The initial unadjusted effect was significant, *F* (1, 342) = 5.89, *P* = .016, with the late group identifying significantly more trauma symptoms (M = 10.52; SD = 5.13) than the early group (M = 8.65; SD = 4.81). However, when the covariates listed above were entered into an ANCOVA with these same independent and dependent variables, the result was not significant, *F* (1, 260) = 3.16, *P* = .077. 

A series of logistic regressions were computed to assess the extent to which a late-term abortion was associated with an increased risk of having met the DSM-IV criteria for the Intrusion, Avoidance, and Hyperarousal subscale scores and for PTSD generally. Although the data presented in Table 3 indicates a higher percentage of late-term abortion respondents meeting the PTSD criteria for the subscales and for the full scale, the results of the logistic regression analyses were not significant.

A second MANOVA and a second MANCOVA were conducted using timing of abortion groups as the independent variable and the 17 items on the PTSD scale as separate dependent variables in an effort to identify specific trauma symptom differences between the two groups. Neither the MANOVA nor the MANCOVA yielded significant multivariate effects. However, several univariate tests were significant in each analysis, and the results are provided in Table 4.

After applying the control variables, the late-term group was significantly more likely to report repeated disturbing dreams, reliving the abortion, and trouble falling asleep. Without the controls, the same items were significant as were feeling emotionally numb, super alert or watchful, and feeling jumpy or easily startled.

### 3.4. Exploratory Analyses

Exploratory analyses were performed to examine the extent to which PTSD scores varied as a function of the reasons that factored into women's abortion decisions. In these analyses, the seven reasons for abortion (mental health, physical health, finances, education, career, family size, and social concerns) listed in Table 2 operated as independent variables. In one test, a MANCOVA, the scores on the three PCL subscale scores served as the dependent variables; whereas in the second test, an ANCOVA, total PCL scores were employed as the dependent measure. All the control variables used in the previous analyses were included in these analyses as well. Due to power concerns, these exploratory analyses could only be conducted with the full sample rather than exclusively focusing on the late-term group. 

The results of the exploratory MANCOVA indicated that only the multivariate effect for social reasons was significant, *F* (3,176) = 4.14, *P* = .007, with the univariate tests for the Intrusion *F* (1,178) = 12.86, *P* = .007, Avoidance *F* (1,178) = 7.70, *P* = .006, and Hyperarousal subscales *F* (1,178) = 4.40, *P* = .007 also significant. In each case, higher means were observed for women who identified social reasons compared to those who did not cite social reasons as relevant to their decisions to abort. Specifically the means on the Intrusion, Avoidance, and Hyperarousal subscales were equal to 1.74 (SE = .26), 3.16 (SE = .34), and 1.74 (SE = .29), respectively, for the group who did not report social reasons as relevant to their decision to abort. In contrast, the means on the Intrusion, Avoidance, and Hyperarousal subscales were equal to 2.99 (SE = .21), 4.41 (SE = .27), and 2.53 (SE = .23), respectively, for the group who did report social reasons as relevant to their decisions. In the ANCOVA with total PCL scores examined, again only the univariate effect for social reasons was significant, *F* (1,178) = 9.04 *P* = .003. The mean PCL total score for the group that did not indicate social reasons were part of their decision to abort was 6.63 (SE = .74); whereas the mean for the group that noted said social reasons were relevant to their decision was considerably higher, 9.93 (SE = .59). Finally, a logistic regression with the same statistical controls employed indicated that women who noted social reasons were relevant to their decision to abort were 186% more likely to experience PTSD symptoms consistent with meeting the DSM-IV diagnostic criteria, OR = 2.86 (CI = 1.57 − 5.23; *P* = .001).

## 4. Discussion

The purpose of this study was to test the hypothesis that women who undergo 2nd and 3rd trimester abortions would be more traumatized than their peers who experience 1st trimester abortions as evidenced by significantly higher rates of PTSD symptoms. After instituting statistical controls for race, marital status, formal education, number of abortions, number of years since the abortion, mental health counseling and hospitalizations for emotional problems before the abortion, meaningfulness of the respondent's religion, and a childhood or adult history of physical or sexual abuse, all the group differences were in the hypothesized direction but only a few were statistically significant. Specifically, the difference in Intrusion subscale scores was statistically significant and when individual PTSD items were examined, the late-term group was found to report more disturbing dreams, more frequent reliving of the abortion, and more trouble falling asleep. The first two of these items are Intrusion subscale symptoms and the last is a symptom from the Hyperarousal subscale. 

Differences between the groups were few due to a large percentage of women from both the early and late abortion groups reporting symptoms of PTSD. In fact, 52.5% and 67.4% in the early and late abortion groups, respectively, met the DSM-IV symptom criteria, a considerably higher percentage than in earlier published reports [28–30]. There are several reasons why these results may have been obtained. First, for this particular sample of women, a great deal of time had elapsed since the abortion (an average of 15 years) and the symptoms could conceivably have developed later in this extended time frame. For example, developmental changes including both normative and nonnormative life events could have been quite stressful and added to the salience of the abortion (e.g., difficulties conceiving a wanted pregnancy, a miscarriage, or relationship problems, etc.). Such events may have triggered a delayed reaction. Second, the fact that the current sample was characterized by high rates of exposure to potentially traumatizing physical and sexual abuse in childhood and adulthood may have yielded a sample with increased susceptibility to experiencing the abortion as a trauma. Third, because the data were collected anonymously and voluntarily, women who had been more negatively affected may have been more interested in the study and were therefore more willing to participate than women who were less adversely impacted. Fourth, in this study the participants were asked if they had experienced the symptoms on the PCL at any point after the abortion and as a result of the abortion, but they were not asked to indicate the symptom duration. Therefore, the high numbers meeting the symptom criteria for PTSD evidenced in this investigation should be viewed cautiously and future studies should incorporate more comprehensive assessments of PTSD symptoms, ideally using clinician administered diagnostic tools. 

Finally, more than a quarter of the current sample had experienced more than one abortion and the effects may have been cumulative. There is evidence that repeated abortion increases the risk for mental health problems [49, 50]. Specifically, Freeman reported that repeated abortion patients exhibited significantly higher distress scores on measures of interpersonal sensitivity, paranoid ideation, phobic anxiety, and sleep disturbance when compared to women who had only one abortion [49]. In that study, somatization, hostility, and psychoticism were likewise elevated in the group of women who had more than one abortion. Similarly, Niemela and colleagues found that Finnish women seeking a second abortion rated lower on impulsivity, emotional balance, realism, self-esteem, life stability, and in the capacity for positive personal relationships compared to women with only one abortion [50]. 

A secondary objective of this study was to identify possible differences in the context of abortion decision-making associated with an early versus late abortion. When compared to a 1st trimester abortion the following differences pertaining to the context of the abortion decision were observed in those who had 2nd or 3rd trimester abortions: (1) the abortion was more likely to have been desired by the partner, (2) the abortion was less likely to have been desired by both parties, (3) there was more pressure from someone other than the partner to abort, (4) male partners were less likely to have been informed of the abortion until afterwards, and (5) women were more inclined to have left the partner before undergoing the procedure. In general, these results are indicative of more ambivalence and conflict surrounding the decision and the likelihood of less stable partner relationships among women who obtain later abortions. Logically, women who are unsure about how to proceed with an unplanned pregnancy are more likely to put off the decision to abort, perhaps hoping that their circumstances will improve and enable them to carry to term.

Other significant differences between the early and late-term groups related to reasons for the abortion. In particular, mental health, financial, and educational concerns figured into decisions to abort more often with 1st trimester abortions compared to later abortions. The only reason that was more frequently reported by the women undergoing later abortions compared to the women who had early abortions was a general category of “physical health concerns” with nearly 30% of the late abortion group voicing these concerns compared to approximately 15% of the early group. Unfortunately no data were gathered pertaining to the specific nature of the concerns and future research should examine the extent to which these factors were based on preexisting conditions or concerns introduced by the pregnancy. 

Interestingly, social concerns such as embarrassment were the most frequently reported concern, with endorsement by 62% of the late term group and 69.1% of the early group. This may have been an additional reason why so many women in the sample experienced stress afterwards as they likely chose the abortion to preserve their reputations despite ambivalence or actually desiring to continue the pregnancy. Some of the women may have also come to feel the abortion was not sufficiently justified. The least commonly identified reason for the choice to abort was family size with approximately 13% of the women in each group indicating this concern entered into their decision. 

The results of the exploratory analyses employing the full sample of women, both those who had early and late abortions, indicated that social reasons for an abortion were strongly related to PCL total scores and to Intrusion, Avoidance, and Hyperarousal subscale scores. With a large percentage of the participants reporting that social reasons entered into their decisions, this could be an additional explanation for why the PTSD rates were so high in the sample. This result has serious implications for counseling prior to an abortion. 

### 4.1. Clinical Implications and Conclusion

These data suggest that women who postpone their abortion into the 2nd or 3rd trimester experience elevated risk for certain forms of unwelcome re-experience of the abortion procedure, likely requiring active professional intervention. Relief from intrusive memories whether in the form of flashbacks or disturbing dreams may only occur if the individual learns to effectively integrate memories of the traumatic experience with other aspects of her past as well as with contemporary experiences and emotions [51]. This is a process that ordinarily involves not only uncovering painful memories, but also transforming them in a personally meaningful way in order to bring relief. Verbalizing traumatic experiences and sharing with others is an essential component of this complex healing process [51] and sadly due to the guilt and related shame which frequently occur in conjunction with an abortion experience [52], many women may never confide in others and are unable to effectively integrate traumatic memories. Over time, the triggers that initiate the intrusion may become more generalized and debilitating as well [51]. For example, a woman may initially experience intrusive symptoms whenever she sees a nurse if most of her interaction was with a nurse during the abortion and then with time, contact with any health care professional in a uniform may initiate an episode of reliving the abortion.

Social reasons for both early and late abortions were related to numerous PTSD symptoms; therefore, professionals should examine the extent to which women are selecting abortions for social reasons, such as to protect their reputation or to avoid embarrassment. In such cases the abortion may, over time, feel unwarranted or frivolous to the woman and incite deep feelings of guilt or remorse that can trigger trauma symptoms. In addition, under such circumstances there is the possibility that the woman desired the pregnancy but decided not to go through with it for social reasons. The research cited previously has shown that pregnancy wantedness is a predictor of postabortion distress. 

Based on employment of a convenience sample, the relatively small number of respondents with late-term abortion experience, and reliance on self-report, this study is most appropriately viewed as a pilot for future analyses of the psychological impact of later-term pregnancy termination procedures. Nevertheless, securing a sample of any size is not an easy prospect when the focus is on late-term abortion and there are very few published reports on this seriously understudied and potentially quite fragile population. Based on societal interest in the morality and legality of late-term abortion, data related to the psychological sequelae of late-term abortion is needed in order to protect women's mental health. 

The results of this study raise serious questions that merit further study. (1) Do women who have later abortions tend to experience intrusive trauma-related symptoms for a longer duration than women who have earlier abortions due to the nature of late-term abortion procedures? (2) Are women with abuse histories who may be primed for trauma more prone to experiencing serious trauma symptoms after an abortion and are they less likely to seek needed help and achieve inner peace due to compromised self-esteem, self-blame, or shame? (3) Are there other mental health problems, such as depression, anxiety, and substance abuse that tend to occur more frequently after later abortion compared to early abortion? (4) Assuming differences are detected and late-term abortions are identified to be more emotionally taxing on women, who are most at risk for mental health problems and what specific characteristics of 2nd and 3rd trimester abortions make them more traumagenic? As long as 2nd and 3rd trimester abortions are medical services that are freely available to women residing in the U.S, we have an ethical obligation to more fully understand the mental health risks involved and to convey this information in a sensitive manner to women as they struggle with difficult abortion decisions.

## Figures and Tables

**Table 1:** Frequencies of demographic and control variable categories for full sample.

Race	
White	85.4%
Black	3.0%
Hispanic	5.7%
Asian	.5%
Other	5.4%

Education at the time of survey	
Less than 12 years	2.7%
High school diploma	21.4%
Technical/associates degree	29.2%
Bachelor degree	28.7%
Graduate degree	18.0%

Marital status at the time of abortion	
Married	14%
Unmarried	86%

Number of children at time of survey	
None	42.0%
One	12.8%
Two	23.0%
Three	13.9%
Four or more	8.2%

Number of abortions	
One	73.4%
Two or more	26.6%

Meaningfulness of respondent's religion	
Not at all	8.2%
Not very	4.4%
Somewhat	10.7%
Important	12.1%
Very important	64.6%

Mental health counseling prior to abortion	
Yes	27.5%
No	72.5%

Hospitalized for emotional reasons prior to abortion	
Yes	3.8%
No	96.2%

Victim of child abuse	
Yes	24.1%
No	75.9%

Victim of sexual abuse in childhood or adolescence	
Yes	36.7%
No	63.3%

Victim of physical abuse during adulthood	
Yes	26.3%
No	73.7%

Victim of sexual abuse during adulthood	
Yes	32.5%
No	67.5%

**Table 2:** Differences in variables surrounding the abortion experience based on abortion timing.

Variables	Early Abortion (up to 12 weaks) *n* = 307	Late Abortion (13–30 weaks) *n* = 52	Significant Differences
Pregnancy desired	28.0%	39.2%	*P* = .106
Pregnancy desired by partner	**10.3%**	**22.4%**	**P** = .018
Respondent and partner supported the decision	**51.9%**	**29.8%**	**P** = .006
Partner pressured for abortion	38.2%	37.5%	*P* = .928
Someone else pressured for abortion	**30.5%**	**47.8%**	**P** = .021
Left partner before decision for abortion was made	**15.6%**	**28.3%**	**P** = .039
Partner did not know about abortion until afterwards	**12.5%**	**23.9%**	**P** = .043
Preabortion counseling received was adequate	14.8%	12.2%	*P* = .637
Information on alternatives was adequate	17.4%	13.7%	*P* = .513
Adequate information was provided on physical and emotional risks	14.8%	9.8%	*P* = .342

Reasons for abortion			
(i) Mental health concerns	**56.8%**	**40.8%**	**P** = .040
(ii) Physical health concerns	**14.7%**	**29.8%**	**P** = .011
(iii) Financial concerns	**70.9%**	**42.9%**	**P** = .0001
(iv) Concern that a child would interfere with education goals	**51.9%**	**34.0%**	**P** = .021
(v) Concern a child would hinder career goals	57.7%	44.0%	*P* = .073
(vi) Did not want a larger family	13.0%	12.5%	*P* = .930
(vii) Concern regarding reactions of others to having a child	69.1%	62.0%	*P* = .323

**Table 3:** Percentage of respondents from the early and late abortion groups meeting PTSD subscale and full scale criteria.

Dependent variables	Early Abortion (up to 12 weaks) *n* = 307	Late Abortion (13–30 weaks) *n* = 52
Met DSM-IV criteria for intrusion	82.1%	91.8%
Met DSM-IV criteria for avoidance	73.3%	76.5%
Met DSM-IV criteria for hyper-arousal	60.1%	67.4%
Met DSM-IV criteria for PTSD generally	52.5%	67.4%

**Table 4:** Results of significant univariate tests for MANOVAs and MANCOVAs performed on individual PTSD items.

PTSD Item	Early Abortion		Late Abortion		*P*-value
	Mean	SD	Mean	SD	
	Mean	SE	Mean	SE	
Disturbing memories, thoughts, images					
Repeated disturbing dreams	.29	.452	.43	.501	*P* = .042
	.30	.031	.48	.078	*P* = .033*
Reliving abortion	.21	.409	.41	.498	*P* = .003
	.22	.028	.41	.070	*P* = .016*
Upset with reminder of abortion					
Physical reaction when reminded of abortion					
Avoided thinking about abortion					
Avoided activities that were reminders of the abortion					
Trouble remembering parts of abortion					
Loss of interest in activities					
Felt distant from others					
Felt emotionally numb	.57	.496	.74	.443	*P* = .029
Felt future would be cut short because of abortion					
Trouble falling or staying asleep	.42	.494	.59	.498	*P* = .032
	.42	.032	.62	.081	*P* = .021*
Irritable or angry outbursts					
Difficulty concentrating					
Super alert or watchful since abortion	.32	.466	.52	.505	*P* = .007
Jumpy or easily startled since abortion	.34	.476	.50	.506	*P* = .040

*Controls for race, marital status at the time of the abortion, number of years of formal education, number of abortions, number of years since the target abortion, having received mental health counseling before the abortion, having been hospitalized for emotional problems before the abortion, meaningfulness of the respondent's religion, and a childhood or adult history of physical or sexual abuse.
